# Dyadic Coping and Illness Uncertainty in Cancer Patient–Caregiver Dyads: Interactive Mechanisms, Heterogeneity, and Integrated Intervention Directions—A Narrative Review (2021–2025)

**DOI:** 10.3390/healthcare14142098

**Published:** 2026-07-14

**Authors:** Ruizhe Cao, Yingchao Zhou, Yanwei Su

**Affiliations:** School of Nursing, Tongji Medical College, Huazhong University of Science and Technology, Wuhan 430030, China; u202313281@hust.edu.cn (R.C.); u202313336@hust.edu.cn (Y.Z.)

**Keywords:** cancer dyads, dyadic coping, illness uncertainty, interactive mechanism, integrated intervention

## Abstract

Cancer patient–informal caregiver dyads function as core collaborative units across the cancer trajectory from diagnosis to recovery. A central challenge for these dyads lies in managing illness uncertainty alongside the demands of dyadic coping, two interrelated factors that jointly shape long-term quality of life, psychological well-being, and disease adaptation. These two factors interact to shape their long-term quality of life, psychological well-being, and disease adaptation. Past studies have mostly examined dyadic coping and illness uncertainty separately. Employing a narrative review design, this study searched the PubMed database for literature published from 2021 to 2025, and synthesizes evidence on association pathways and moderating factors between dyadic coping and illness uncertainty among adult cancer patient–informal caregiver dyads. Existing research on dyadic coping has predominantly focused on the effects of interventions (e.g., enhanced spousal communication, joint exercise) on emotional health and relationship quality. Studies on illness uncertainty have largely examined its association with anxiety and depression, but have failed to provide a comprehensive understanding of the interactive pathways between the two constructs. This review addresses this gap by synthesizing evidence from three perspectives: interactive mechanisms, heterogeneous characteristics, and integrated intervention directions. Evidence from the reviewed literature suggests that illness uncertainty represents a critical antecedent of dyadic coping among cancer dyads. Illness uncertainty and dyadic coping share a bidirectional association, and this relationship is moderated by factors including cancer type, patient age, and cultural background. Most uncertainty management interventions effectively reduce illness uncertainty in specific populations. Synthesized evidence indicates that dyadic coping is a significant predictor of relationship satisfaction among couples coping with chronic illness. Interventions that are made for these couples need to include things like psychoeducation and skill-building parts that are about dyadic coping, so that they can improve the couples’ relationship satisfaction. Based on predominantly observational evidence with heterogeneous study designs, the authors propose that combining uncertainty management interventions with dyadic coping skills training represents a key direction for future clinical care optimization. This review provides evidence-based implications for developing targeted dyadic care strategies and advancing family-centered, full-course cancer care models.

## 1. Introduction

Cancer has become a global public health priority, imposing persistent physical, psychological, and social distress on patients and family caregivers [1]. As a chronic, progressive, and prognostically ambiguous disease, cancer places patient-caregiver dyads under long-term stress. These dyads constitute the core supportive unit throughout diagnosis, treatment, and rehabilitation, and their mutual adaptation is closely associated with quality of life, psychological resilience, and disease outcomes. Illness uncertainty and dyadic coping are widely regarded as two core challenges for cancer dyads, and their interaction is thought to influence adjustment processes, yet relevant joint research remains limited [2,3]. This line of inquiry is grounded in core theoretical frameworks including Mishel’s Uncertainty in Illness Theory and the Actor-Partner Interdependence Model, which jointly explain individual and interpersonal adaptation processes within cancer dyads. Clarifying their interactive mechanisms and integrated intervention value is urgently needed to guide person-centered and family-focused cancer care [4,5,6].

Studies have shown that patient-spouse dyads facing cancer jointly cope with physical and psychological stress. Emotion expression and communication patterns driven by attachment styles significantly affect disease adaptation; these two factors serve as core interpersonal manifestations and important predictors of dyadic coping among couples affected by cancer [7]. Uncertainty surrounding cancer is particularly pronounced in disparities of incidence and mortality associated with race, age, and gender. Dyadic coping requires the integration of individual prevention and population health equity, and integrated interventions must focus on the popularization of screening and equity in treatment to respond effectively. Emerging digital and virtual reality-assisted cognitive behavioral interventions have demonstrated the value of integrated mental health models in chronic disease management, providing a reference framework for optimizing dyadic interventions in cancer care.

Cancer-related cachexia is highly prevalent across disease trajectories, particularly in end-stage populations, and adds complexity to clinical supportive care. Integrated psychoeducational and rehabilitation interventions designed for patient-caregiver dyads remain insufficiently investigated, as evidenced by ongoing feasibility trials in this area [8]. The fluctuating physical symptoms, functional decline and ambiguous prognosis associated with cachexia further amplify perceived illness uncertainty among both patients and caregivers, which in turn disrupts coordinated dyadic coping processes. Illness uncertainty represents a persistent experience for cancer patient-caregiver dyads throughout diagnosis, treatment, and rehabilitation. Among older adults with advanced cancer, higher levels of illness uncertainty are positively correlated with anxiety, depression, and distress, and negatively associated with quality of life and emotional well-being [9]. For family caregivers of patients with advanced lung cancer, perceived illness uncertainty is significantly positively correlated with anticipatory grief, and caregiver burden is an independent influencing factor of anticipatory grief [10].

Accumulating evidence suggests that illness uncertainty may reduce information communication efficiency within dyads, while inadequate dyadic coping may further amplify perceived uncertainty and form a vicious cycle impairing adaptation, though this cyclical model has not been consistently validated across all cancer populations. Targeting only uncertainty or only dyadic coping is insufficient to improve holistic outcomes. Separate systematic reviews focused on chronic disease populations have identified bidirectional pairwise correlations between anxiety, depression, physical functionality and health-related quality of life. Cross-sectional evidence indicates impaired physical function and reduced quality of life significantly coincide with greater emotional distress, while elevated anxiety and depressive symptoms are associated with diminished daily functional capacity and overall well-being—a consistent pattern observed across various chronic conditions including lung and breast cancer subtypes [11]. Therefore, exploring their interactive mechanisms and developing integrated interventions carry important theoretical and clinical significance for promoting full-course and family-centered cancer management [12,13,14].

A qualitative study of 39 female partners of prostate cancer patients in Denmark confirmed that community-based football interventions can significantly improve patients’ conditions, yet fail to adequately address the partners’ psychological and caregiving support needs. The lack of dyadic collaborative design in the intervention represents one important reason why the partners’ unmet and often invisible needs remain unaddressed [15]. A 2023 Chinese study reporting an APIM analysis among 443 lung cancer survivor–spouse dyads indicated that patients’ unmet supportive care needs (measured by the SCNS scale) not only impaired their own quality of life (assessed by the WHOQOL-BREF scale) but also exerted a negative impact on their spouses’ quality of life via the partner effect [16]. These findings contribute to a comprehensive understanding of the linkage mechanisms between the two constructs and the development of integrated intervention strategies, holding important practical implications for improving the quality of care for cancer dyads.

While prior narrative reviews have examined dyadic coping or illness uncertainty separately in oncology populations, no recent systematic synthesis has integrated the bidirectional interactive mechanisms between the two constructs and their heterogeneous manifestations across cancer contexts. Although research on cancer dyadic coping and illness uncertainty has made some progress between 2021 and 2025, several critical limitations remain. First, research perspectives are fragmented: many studies examine dyadic coping or illness uncertainty in isolation, ignoring their interactive effects. For example, an exploratory controlled study from Belgium tested a group-based self-hypnosis and self-care intervention delivered to female cancer patients. It evaluated outcomes of dyadic illness communication and dyadic coping between patients and their partners, but did not include illness uncertainty as an observed or analyzed variable [17]. A 2022 longitudinal study enrolled 397 dyads of older adults with advanced cancer and their caregivers in the United States. It confirmed significant actor and partner effects of illness uncertainty between the two parties, but did not examine the mediating or moderating effects of dyadic coping [18]. Interventions lack sufficient targeting: most current interventions focus on only one domain (e.g., improving communication alone or reducing uncertainty alone) and lack a comprehensive design addressing their combined effects. The ‘Me in We’ dualistic communication intervention published in the United States in 2021 has demonstrated feasibility and acceptability among patients with advanced cancer and their caregivers. It can promote communication, goal sharing, and relationship satisfaction between the two groups. Similarly, the MGCS mobile app intervention (published 2023) in China significantly reduced illness uncertainty among patients with gynecologic cancer undergoing chemotherapy at the 24-week follow-up [19], yet did not adopt a caregiver-inclusive perspective and therefore could not establish a long-term mechanism for dyadic collaborative coping. Insufficient attention has been paid to heterogeneity: studies examining differences across cancer types and population characteristics (age, cultural background) remain fragmented and lack in-depth and integrated investigation. Regarding cancer type heterogeneity, existing research has focused predominantly on breast and prostate cancer; there is a dearth of qualitative research on dyadic coping between pancreatic cancer patients and their spouses, leaving this field relatively under-explored [20]. Metastatic uveal melanoma, as a rare tumor, has limited research on patient uncertainty in the era of immunotherapy and targeted therapy [21]. In China, lung cancer patient–spouse dyads in Fujian province are influenced by collectivist culture, in which spouses typically serve as primary caregivers. Relevant findings suggest that tailored supportive care interventions should be designed for these dyads [16]. Limitations exist in measurement tools: there is a lack of comprehensive assessment instruments that simultaneously cover both dyadic interaction and illness uncertainty. Accordingly, this review addresses three key unanswered questions: (1) the core interactive pathways between dyadic coping and illness uncertainty in cancer dyads; (2) the moderating effects of heterogeneous characteristics; and (3) core components of integrated dual-target interventions. Meanwhile, the cross-cultural adaptability and dynamic assessment capacity of existing tools remain inadequate. For example, a 2021 Chinese psychometric evaluation of the revised Dyadic Adjustment Scale noted that while the scale demonstrated acceptable reliability and validity, its sample was restricted to non-religious rural populations in Shanxi Province, and no clear cutoff scores were established [22]. A study used a researcher-adapted short-form version of the Adult Illness Uncertainty Scale to reduce burden among patients with advanced cancer and severe symptoms and their family caregivers, as well as to assess their respective levels of illness uncertainty. However, this adapted scale has not been validated, and further examination of its psychometric properties is warranted in this and other populations [23].

Given the broad scope of this topic spanning interactive mechanisms, heterogeneous characteristics and intervention frameworks, a narrative review was adopted to enable flexible, concept-driven synthesis of diverse study designs, which is more suitable for mapping the research landscape and proposing integrated theoretical frameworks. The strength of this narrative review lies in synthesizing recent international evidence. It emphasizes the analysis of heterogeneity across multiple dimensions, including cancer type, population characteristics, and disease stage, and proposes an integrated dual-core intervention framework of uncertainty cognitive restructuring and dyadic coping skills training. The core content is organized around several aspects: the evolution of core concepts and measurement tools, interactive mechanisms, heterogeneous manifestations, intervention practices and innovative frameworks, as well as future research directions and clinical translation pathways.

## 2. Methods

This narrative review was conducted in accordance with the Scale for the Assessment of Narrative Review Articles (SANRA) guidelines (a widely accepted framework for narrative review reporting) and standard conventions for oncology care research. PubMed was selected as the sole database as it is the most comprehensive and authoritative source for peer-reviewed oncology and nursing research, covering the majority of high-quality international studies in this field. We acknowledge that this single-database approach may omit studies indexed exclusively in other databases (e.g., Embase, CINAHL), which may limit the comprehensiveness of the review; this limitation is further elaborated in the Limitations section.

### Full Search Strategy

The search strategy combined both MeSH terms and free-text keywords, and the literature search was conducted on 31 March 2025, covering publications from 1 January 2021 to 31 March 2025. The full search string combined terms with Boolean operators AND/OR: ((“Neoplasms” [MeSH Terms] OR “Cancer” [Title/Abstract] OR “Carcinoma” [Title/Abstract] OR “Tumor” [Title/Abstract] OR “Oncology” [Title/Abstract] OR “Malignancy” [Title/Abstract]) AND (“Uncertainty” [MeSH Terms] OR “Illness Uncertainty” [Title/Abstract] OR “Perceived Uncertainty” [Title/Abstract] OR “Disease-related Uncertainty” [Title/Abstract] OR “Mishel Uncertainty” [Title/Abstract]) AND (“Dyadic Coping” [Title/Abstract] OR “Dyadic Adjustment” [Title/Abstract] OR “Spousal Coping” [Title/Abstract] OR “Partner Coping” [Title/Abstract] OR “Couple Coping” [Title/Abstract] OR “Interpersonal Coping” [Title/Abstract] OR “Dyadic Intervention” [Title/Abstract] OR “Dyadic Relationship” [Title/Abstract] OR “Common Coping” [Title/Abstract] OR “We-disease” [Title/Abstract])).

No additional handsearching of reference lists was performed to maintain scope consistency.

Filters were set to include only English articles published between 1 January 2021 and 31 March 2025. This time frame was selected to focus on the most recent evidence reflecting contemporary cancer care models. The COVID-19 pandemic has substantially altered care delivery patterns and dyadic coping experiences since 2020, and studies from 2021 onward capture these emerging trends. Foundational theoretical studies published before 2021 are cited as conceptual background but not included in the formal evidence synthesis.

After removing duplicates, two reviewers independently screened titles, abstracts, and full texts. Disagreements on eligibility or data extraction were resolved by discussion between the two reviewers; if consensus was not reached, a third senior reviewer made the final decision. The study selection process followed PRISMA 2020 guidelines, with the number of records at each screening stage reported as follows. Overall, 218 records were initially identified from PubMed. After removing 50 duplicates, 168 records were screened by title and abstract, with 49 excluded for ineligibility. Full texts of 119 records were further assessed for eligibility, among which 40 articles were excluded (21 review articles and 19 research studies), and 79 studies were finally included in the narrative synthesis. A PRISMA-compliant flow diagram of the study selection process is provided as Supplementary Materials. Eligible studies met the following criteria: (1) participants were adult (≥18 years) cancer patients and their informal family caregivers; (2) investigated the association, mechanisms or interventions of dyadic coping and illness uncertainty; (3) reported quantitative outcomes, qualitative findings, scale psychometric properties, or methodological/theoretical frameworks; (4) full-text articles published in English, including original research, systematic reviews/meta-analyses, and targeted review articles; (5) conducted in clinical or community settings.

Original empirical studies constitute the core evidence base of this review; review articles are included only for auxiliary purposes such as research landscape mapping and cross-study comparison, and do not serve as direct evidence for core conclusions.

Exclusion criteria included non-English publications, conference abstracts, letters, editorials, case reports, study protocols, studies focusing on pediatric cancer or professional caregivers, as well as reviews and research studies that failed to meet the eligibility requirements. Review articles that did not meet the thematic scope or eligibility criteria were excluded during full-text assessment; eligible review works were retained in the final evidence synthesis to complement empirical findings, with their use strictly limited to background and comparative discussion. A small number of out-of-scope publications were only cited as background contextual references and did not participate in evidence synthesis.

Prior to narrative synthesis, the methodological quality of included studies was appraised using the Joanna Briggs Institute (JBI) critical appraisal checklists corresponding to each study design, to inform the grading of evidence strength and interpretation of findings. All 79 eligible studies were included in the final narrative synthesis.

Data were extracted using a standardized form and synthesized narratively around five themes: concepts and measurements, interactive mechanisms, heterogeneity, interventions, and future directions. No quantitative meta-analysis was performed. The 79 included studies were categorized by study type: 18 intervention studies, 24 cross-sectional studies, 15 observational studies, 10 systematic reviews/meta-analyses, 6 qualitative studies, 4 review articles, and 2 methodological/theoretical studies.

Original research articles (intervention, cross-sectional, observational, qualitative and methodological studies, n = 65) formed the core evidence base for synthesizing interactive mechanisms, heterogeneity and intervention effects; review articles (n = 14) were used only to contextualize the research background and compare findings with prior syntheses.

## 3. Core Concepts and Measurement Development of Dyadic Coping and Illness Uncertainty

Overall, 47 studies were included, comprising 22 cross-sectional studies, 11 qualitative studies, 7 longitudinal cohorts, 5 randomized controlled trials, and 2 methodological validation studies. Studies were mainly conducted in China, the U.S. and European countries, with sample sizes ranging from 10 to 722 dyads. Breast cancer, lung cancer and prostate cancer were the most studied cancer types, and most dyads were spousal pairs.

### 3.1. Definition and Theoretical Foundations of Cancer Dyadic Coping

Cancer dyadic coping refers to the dynamic process in which cancer patients and their partners jointly respond to stressful events by providing mutual support, communicating about stressors, and collaboratively adjusting their coping behaviors when confronted with illness-related stress [24].

Its core theoretical foundations include the Actor–Partner Interdependence Model (APIM) and the ABC-X Family Stress Model. Two core theoretical frameworks underpin research on cancer dyadic coping: the Actor–Partner Interdependence Model (APIM) and the ABC-X Family Stress Model. The APIM conceptualizes how individual characteristics in a dyad exert both actor effects (influencing one’s own outcomes, estimated while controlling for the partner’s variables, distinct from individual-level coping mechanisms that ignore dyadic interdependence) and partner effects (influencing the other member’s outcomes), serving as the primary analytical framework for dyadic data. The ABC-X Family Stress Model explains how family systems adapt to stressful events, emphasizing the interplay of stressor severity, available resources, and cognitive appraisal in shaping coping outcomes. The APIM indicates that relationship satisfaction (as an antecedent variable) between pancreatic cancer patients and their spousal caregivers influences their own anxiety and depression through their individual dyadic coping (actor effects), and patients’ relationship satisfaction also affects spouses’ anxiety and depression via the spouses’ dyadic coping (unidirectional partner effects) [25].

Based on the ABC-X Family Stress Theory and the Actor–Partner Interdependence Mediation Model (APIMeM), this study found that dyadic coping between breast cancer patients and their spouses affects family adaptation through the mediating role of benefit finding, with significant actor effects and unidirectional partner effects [26].

Based on the nature and direction of coping strategies, dyadic coping can be divided into three core dimensions: first, positive dyadic coping, including supportive, delegated, and joint dyadic coping [27]; second, negative dyadic coping, involving hostile, ambivalent, or superficial responses such as blaming one’s partner for inadequate stress management [28]; third, empowered dyadic coping, which refers to reducing a partner’s stress by taking over part of their tasks and responsibilities [29]. A 2022 Dutch multicenter eQuiPe study of 566 advanced cancer patient–partner dyads showed that negative dyadic coping was the most frequently used, while joint dyadic coping was the least common. Moreover, patients reported significantly higher satisfaction with dyadic coping than their partners, indicating perceptual differences in coping within the dyad [28].

### 3.2. Cancer Illness Uncertainty

#### 3.2.1. Conceptual Definition and Theoretical Foundation

Cancer illness uncertainty refers to the cognitive state in which individuals cannot accurately predict disease outcomes due to insufficient access to or interpretation of illness- and treatment-related information (such as treatment efficacy and prognosis) [9]. An important theoretical base for studies about illness uncertainty is the ‘Uncertainty in Illness Theory’ that Mishel put forward in the 1980s. This theory forms the basic starting point for understanding how patients experience illness uncertainty and adapt to it, and it focuses mostly on how individuals process uncertainty inside their own minds.

#### 3.2.2. Empirical Evidence on Sources and Impacts of Illness Uncertainty

During clinical consultations for advanced pediatric cancer, discussions involving clinicians, parents, and patients address six distinct categories of uncertainty-related topics. These groups are how suitable a treatment is, short-term harmful side effects and health issues, what will happen with the illness or how the treatment will work, uncertainty about the diagnosis, uncertainty about practical arrangements for care, and harmful side effects that show up after a long time [30].

Why people get uncertainty when they have cancer is connected to many different things. If we look at it from a medical point of view, using modern precision medicine tools like polygenic testing has made diagnosis and treatment more accurate. But all the extra genetic information that these tests produce has actually made the related uncertainty even bigger than before [31]. When looking at the level of individual patients, how much health knowledge they understand and how good they are at looking up health information both affect how uncertain they feel about their own situation. For example, patients who do not have much health knowledge often feel more worried because they cannot fully understand what the treatment plans mean for them [32]. On a broader social level, public health events like global pandemics can make uncertainty even worse. One clear example is that the COVID-19 pandemic led to delays for many cancer surgeries, which made patients more worried about how their disease would get worse over time [33]. A 2021 qualitative study from the U.S. looked at 35 outpatient audio recordings from meetings between doctors and kids with late-stage pediatric cancer. It found that 63% of all statements about uncertainty were started by the doctors themselves, and “whether treatment is right” was the topic that came up more than any other. Pediatric patients rarely participated in these uncertainty-focused discussions, indicating an imbalance in communication participation between parties [30].

### 3.3. Development and Limitations of Measurement Tools

#### 3.3.1. Measures of Dyadic Coping

Existing dyadic coping measures differ in core focus: the Dyadic Coping Inventory (DCI) emphasizes stress communication processes and specific coping behaviors, while the Revised Dyadic Adjustment Scale (R-DAS) focuses on overall relationship quality and dyadic consensus; the former is more widely used in intervention research, while the latter is more common in cross-sectional observational studies.

Between 2021 and 2025, the primary measurement tools for dyadic coping were the Dyadic Coping Scale and its short form, supplemented by context-specific scales such as the Relationship Closeness Scale and the Dyadic Adjustment Scale. The Dyadic Coping Scale, developed by Bodenmann in 2008, assesses mutual support and assistance between couples when facing stressful events. It covers multiple dimensions including stress communication, positive dyadic coping (encompassing supportive, delegated, and joint coping), negative dyadic coping, and perceived effectiveness of couples’ stress management, while also incorporating evaluations of marital satisfaction. In the French version, Cronbach’s α values ranged from 0.64 to 0.89, with the subscale for negative dyadic coping yielding α values of 0.50 to 0.53. In the sample used in the present study, the scale demonstrated Cronbach’s α values ranging from 0.75 to 0.93, and the negative dyadic coping subscale had α values of 0.62 to 0.76 [24].

Progress has also been made in scale development for specific populations. In 2021, a Chinese study translated the Revised Dyadic Adjustment Scale (R-DAS) into Chinese and evaluated its psychometric properties among 126 gynecologic cancer patient–partner dyads. The results showed that the overall Cronbach’s α coefficient of the scale was 0.85, the test–retest reliability (ICC) was 0.88, and scores were significantly positively correlated with the Quality of Marriage Index (QMI), filling the gap in Chinese-language dyadic relationship assessment tools for this population in mainland China [22]. Although the Dyadic Coping Inventory (DCI) was designed for general stress, it has been validated for use among patients with advanced cancer and their partners. The 37-item inventory includes dimensions of common, supportive, and negative coping, with scores standardized to a 0–100 scale to distinguish levels of coping. In a study of 566 couples in this population, the instrument detected weak to moderate interdependence in perceived coping between partners (r = 0.27–0.56). APIM analyses revealed significant associations, notably that partner-reported satisfaction with dyadic coping was positively linked to emotional functioning in both patients and partners, highlighting the dyadic nature of adjustment in this population [28].

#### 3.3.2. Measures of Illness Uncertainty

The Mishel Uncertainty in Illness Scale (MUIS) family is the dominant measurement system, including full versions, short forms, and caregiver-specific adaptations. These instruments share the same theoretical foundation and differ mainly in length, item coverage, and target population. Measures of illness uncertainty are dominated by the ‘Mishel Uncertainty in illness Scale (MUIS)’ family, including the full 32-item version, short forms, and population-specific adaptations (e.g., for older adults and caregivers). The 9-item short-form MUIS uses a 5-point Likert scale ranging from “strongly disagree” to “strongly agree,” with higher scores indicating greater uncertainty [9]. The development of scales for specific scenarios has also been gradually advanced. In a Dutch study on cancer genetic counseling published in 2021, a self-designed questionnaire was employed to measure feelings of uncertainty [31]. A 2022 Chinese study among family caregivers of patients with advanced lung cancer indicated that the ‘Uncertainty in illness Scale (Family Caregiver Version)’, which contains 30 items, can effectively evaluate the level of illness uncertainty among caregivers [10].

#### 3.3.3. Common Limitations of Existing Instruments

Despite continuous improvements in measurement tools, three key limitations remain: First, insufficient integration: there is still no assessment instrument that covers both dyadic coping and illness uncertainty simultaneously, making it impossible to measure their interactive effects synchronously. For example, existing studies on patients with advanced cancer and their family caregivers suffer from inadequate assessment tools (e.g., unvalidated short-form illness uncertainty scales and no latent variable modeling for the multidimensional structure of quality of life). Moreover, previous studies have not analyzed the interactive effects between illness uncertainty and quality of life using the patient–caregiver dyad as the unit of analysis [23].

Second, cross-cultural adaptability needs to be improved. Most scales are developed based on Western culture and require further validation for applicability in Eastern cultural contexts. For instance, the 2021 Chinese R-DAS study showed that among rural, non-religious participants, the item–total correlation coefficient for the ‘religious matters’ item under the consensus dimension was relatively low (0.246), indicating that this item needs further validation in Chinese regions with greater religious diversity [22].

Third, there is a lack of dynamic assessment functions. Most existing scales are cross-sectional and cannot capture the developmental trends of dyadic coping and uncertainty during disease progression. For example, a 2022 longitudinal qualitative study in Sweden of 11 patients with metastatic castration-resistant prostate cancer found that patients experience uncertainty in interpreting symptoms and signs as their disease progresses [34].

These measurement gaps directly constrain the synchronous assessment of dyadic coping and illness uncertainty and partly explain the inconsistent effect estimates across included studies.

## 4. Interactive Mechanisms Between Dyadic Coping and Illness Uncertainty

**Figure 1:**
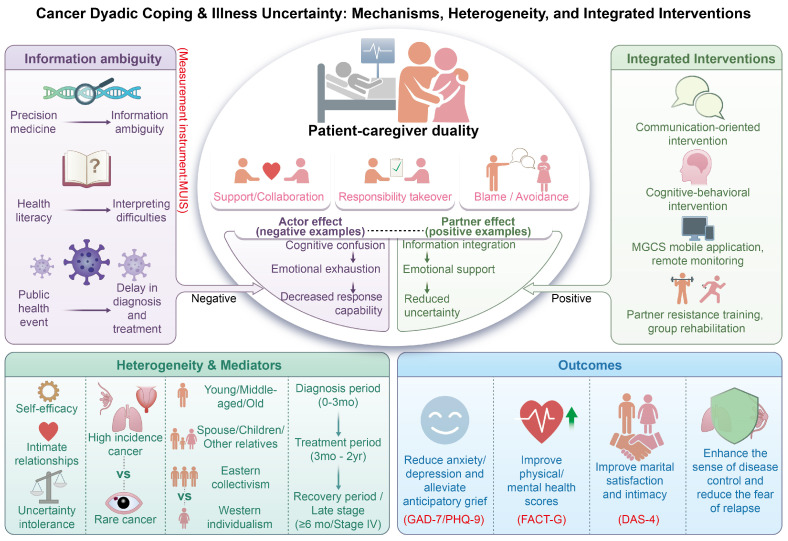
Conceptual framework of dyadic coping and illness uncertainty in cancer patient–caregiver dyads. (**Left panel**): sources of illness uncertainty (measured by the MUIS). (**Center**): three dyadic coping patterns with negative actor-effect and positive partner-effect pathways. (**Upper right**): four integrated intervention approaches. (**Lower left**): heterogeneity dimensions and mediating variables. (**Lower right**): psychological, health, relational and cognitive outcomes.

The interactive pathways, moderating factors and intervention directions synthesized in this review are visually summarized in Figure 1. Note on evidentiary strength: Evidence in this section comes from study designs of varying rigor. In descending order of strength: randomized controlled trials (high), prospective longitudinal studies (moderate-high), cross-sectional studies (moderate), qualitative studies (low-moderate, for exploratory insights), and case reports (low, only for contextual illustration). When synthesizing conflicting findings, we prioritized evidence from larger samples, more rigorous designs, and replicated results across populations. Findings from small single-site studies were marked as preliminary and requiring further validation. The interactive mechanisms described in this section represent conceptual inferences drawn from the synthesized empirical evidence; given the predominantly observational design of included studies, definitive causal relationships cannot be established.

### 4.1. Bidirectional Influence Pathways

#### 4.1.1. Negative Driving Effect of Illness Uncertainty on Dyadic Coping

Cross-sectional and qualitative evidence suggests that illness uncertainty is associated with weakened positive dyadic coping capacity and exacerbated negative coping through the pathway: ‘cognitive confusion-emotional exhaustion-reduced coping ability’. Specific manifestations include:

**Information ambiguity may inhibit positive dyadic communication.** This pathway is indirectly supported by evidence linking unmet information needs and ambiguous medical information to poorer communication quality and increased negative interaction within dyads. When medical information is incomplete or contradictory, dyads tend to reduce effective communication due to cognitive confusion. Such communication barriers and unmet information needs further link to poor dyadic outcomes. Cross-national evidence consistently supports this pathway: among advanced cancer patient-caregiver dyads in the U.S., hostile communication patterns among caregivers were positively associated with their subjective burden, while constructive problem-solving discussions predicted better caregiver preparedness [35]. In South Korean cancer patient-family dyads, unmet information needs consistently predicted poorer physical and mental quality of life for both members, though cross-partner effects were not detected [36].

**Treatment complexity may reduce dyadic coping synergy.** When disease prognosis is highly uncertain, dyads tend to develop helplessness and further adopt negative coping strategies such as avoidance and blame. This association between psychological distress and maladaptive coping behaviors is not restricted to cancer dyads; a post-pandemic cross-sectional study of Portuguese university students confirmed that higher overall levels of depression, anxiety, and stress were significantly associated with an increased likelihood of hazardous alcohol consumption [37]. A 2024 Chinese qualitative study of 10 pancreatic cancer patient–spouse dyads showed that, given the 5-year survival rate of pancreatic cancer of only 11%, some patients and their spouses engaged in silent and avoidant negative coping behaviors, such as avoiding discussing the disease [20]; this exploratory finding requires validation in larger, more diverse samples. A 2022 Dutch multicenter eQuiPe study of 566 advanced cancer patient–partner dyads (using the DCI) found that negative dyadic coping was the most frequently used coping style among both partners, with moderate interdependence in how patients and partners perceived it (r = 0.44, *p* < 0.001) [28].

**Negative dyadic coping may reinforce the uncertainty cycle.** When treatment plans are really complicated, like when patients have multiple rounds of chemo plus radiation therapy together, pairs of patients and caregivers often end up with messed-up role splits, and this hurts how well they can handle things together. One study showed that in situations like this, caregivers often get depressive symptoms because stress builds up over time. When a caregiver has depression, it does not just make how close they feel to the patient smaller; it also makes the emotional connection the patient feels weaker (b = −0.054, *p* = 0.004; b = −0.041, *p* = 0.011). Because intimacy works as one important protective thing that helps two people cope with illness together, when it gets damaged, that ability to work together on coping gets even smaller [38]. One other study showed that for cervical cancer patients getting combined chemoradiotherapy, those who got more than four rounds of chemotherapy or had low dyadic coping had a higher chance of falling into the group with high symptom burden [39]. This negative effect that drives poorer decisions is especially clear when people have to make critical treatment choices for some specific kinds of cancer. When people with peritoneal cancer and their family caregivers have to decide whether to have palliative surgery, the uncertainty about their illness makes it much harder to make a good decision. On top of that, there is often not enough clear and steady back-and-forth communication with the medical team, and they also do not understand enough about how surgery will change their daily life and control their symptoms. All this makes it even harder for the patient and caregiver pair to cope with the situation in a good way. It may even exacerbate distress when treatment goals are misaligned with personal needs [40].

#### 4.1.2. The Moderating Effect of Dyadic Coping on Illness Uncertainty

As a key form of interpersonal emotion regulation, dyadic coping shows small to moderate positive correlations with improved quality of life and reduced depressive symptoms among cancer survivors. Several studies have framed this relationship using theoretical frameworks such as the dyadic illness management theory [41]. Positive dyadic coping is associated with reduced perceived uncertainty within the dyad through the pathway of ‘information integration-emotional support-strategic coordination’, whereas negative dyadic coping intensifies uncertainty. Specific manifestations include:

**Positive joint coping reduces information ambiguity.** By jointly seeking information and integrating medical advice, dyads can alleviate information confusion and lower uncertainty. A 2021 U.S. intervention study titled “Me in We” on bidirectional communication revealed that after two sessions of shared goal discussion (approximately one month apart) among 13 advanced cancer patient–caregiver dyads, participants reported high overall satisfaction with the discussions, strong feelings of being understood, and all completed the study with no attrition [42]. A 2022 Canadian online intervention study (Couplelinks) of 30 young adult breast cancer patient-partner dyads found that following participation in an intervention consisting of six dyadic learning modules, couples reported an overall satisfaction rating of 4.3 out of 5. Most participants considered professional guidance necessary and reported improved communication and enhanced relational solidarity [43].

**Supportive dyadic coping alleviates emotional uncertainty.** Emotional support from caregivers to patients and patients’ empathy for caregivers’ burden can relieve anxiety caused by uncertainty and thereby reduce subjective perceptions of uncertainty. A cross-sectional study of 206 spouses of cervical cancer patients in China showed that self-efficacy (measured by the GSES) played a partial mediating role in the relationship between dyadic coping (DCI) and quality of life (SF-12), with the mediating effect accounting for 16% of the total effect [27]. A 2023 three-arm randomized controlled trial in the United States involving 67 glioma patient–caregiver dyads found that caregivers in the individual yoga (CY) intervention reported significantly greater subjective benefits than those in the dyadic yoga (DY) group, experienced less caregiving-related health decline compared with the DY group, and showed a moderate improvement in mental quality of life relative to the usual care (UC) group (d = 0.46) [44].

**Negative dyadic coping reinforces the cycle of uncertainty.** Avoidant communication, overprotection, and other negative coping behaviors can lead to information isolation and further escalate uncertainty. A 2021 Belgian comparative study of 55 female cancer patient–male partner dyads showed that an 8-week intervention of self-hypnosis combined with self-care delivered only to patients yielded no significant group-by-time interaction effects on emotional distress, dyadic communication, or dyadic coping for either partner. Moreover, patients’ and partners’ perceptions of dyadic communication and dyadic coping were positively correlated [17]. A 2022 Chinese APIM analysis of 182 postoperative lung cancer patient–caregiver dyads found that caregiver depression exerted negative effects on both their own intimacy and the patient’s perceived intimacy. In addition, depression and intimacy were positively correlated within both patients and caregivers, respectively [38].

### 4.2. Key Mediating and Moderating Factors

#### 4.2.1. Mediating Factors: “Bridge Variables” Connecting Dyadic Coping and Illness Uncertainty

Self-efficacy, defined as an individual’s confidence in their own coping ability, acts as a mediating variable between dyadic coping and illness uncertainty. A Chinese study of 206 spouses of cervical cancer patients revealed that dyadic coping (measured by the DCI) exerted a direct positive effect on quality of life and an indirect positive effect through self-efficacy (measured by the GSES), with the mediating effect of self-efficacy accounting for 16% of the total effect [27].

Studies focusing on cervical cancer patient-spouse dyads have indicated that self-efficacy plays a complete mediating role in the link between dyadic coping and MCS, while serving as a ‘partial mediator’ in the link between dyadic coping and physical health-related quality of life (PCS). Additionally, spouses exhibited significantly higher levels of both dyadic coping and self-efficacy compared with the patient group [45].

A 2023 survey of 112 elderly lung cancer patients receiving anticancer treatment in South Korea found that both self-efficacy and uncertainty appraisal (danger/opportunity) were influencing factors of quality of life (FACT-L scale), and together with type and frequency of anticancer treatment, explained 74.2% of the variance [46].

Intimate relationship: the strength of emotional bonding within the dyad, which moderates the effect of dyadic coping on uncertainty. An Actor-Partner Interdependence Mediation Model (APIMeM) analysis among breast cancer patient–spouse dyads in China showed that dyadic coping (C-DCI scale) demonstrated a full actor-mediated effect on quality of life (SF-36 scale) via intimate relationship (Marital Adjustment Test, MAT) [47].

Intolerance of uncertainty: an individual’s level of acceptance of uncertainty, which mediates the relationship between illness uncertainty and psychological outcomes. A cross-sectional study of 108 patients with stage III/IV cancer in the United States, with data collected from 2018 to 2019 and published in 2024, revealed that intolerance of uncertainty directly affected anxiety and depressive symptoms and indirectly influenced them through experiential avoidance; the indirect effect on anxiety was significant at moderate to high levels of trust in physicians [48]. A Chinese study of 202 newly diagnosed cancer patients, with data collected in 2023 and published in 2024, found that intolerance of uncertainty (IU) played a partial mediating role between illness uncertainty (UI) and fear of cancer progression (FoP), accounting for 47.6% of the total effect [49].

#### 4.2.2. Moderating Factors: “Boundary Conditions” Affecting the Strength of the Relationship Between the Two Variables

**Cancer type:** Differences in disease trajectories across cancer types moderate the association between dyadic coping and uncertainty. A 2023 latent profile analysis of 254 breast cancer patient–spouse dyads in China identified four distinct dyadic coping subgroups. The high-level dyadic coping subgroup scored significantly higher than the other three subgroups on all dimensions of the Dyadic Coping Inventory (DCI) and also achieved significantly higher scores on growth (PTGI) [50].

**Age and life cycle:** The age of the dyad and family life cycle influence coping capacity and perceived uncertainty. A 2024 qualitative study of 15 young adult cancer couples in the United States found that they faced challenges including dyadic communication and relationship changes, and that cancer-related uncertainty could trigger negative communication and psychological distress [51]. A 2022 U.S. longitudinal study of 397 late-life cancer patient–caregiver dyads aged 70 years and older revealed that perceived uncertainty decreased over time for both members of the dyad. Caregivers in the geriatric assessment (GA) intervention group showed a greater reduction in uncertainty than those in the usual care group, and uncertainty levels of patients and caregivers were mutually influential [18].

**Cultural background:** Family role divisions under different cultures influence the patterns and effectiveness of dyadic coping. An APIM analysis of 443 lung cancer survivor–spouse dyads in China (2020–2021) showed that unmet supportive care needs of both partners exerted significant negative actor effects on their own quality of life. Survivors’ unmet needs also had a negative partner effect on spouses’ quality of life, whereas spouses’ unmet needs had no significant effect on survivors’ quality of life [16].

**Disease stage:** Differences in the stage of cancer progression influence the sources of uncertainty and the focus of dyadic coping. A cross-sectional study conducted in China from November 2022 to April 2023 among 316 patients with stage III/IV advanced lung cancer revealed that their illness uncertainty comprised three dimensions: ambiguity, lack of clarity, and unpredictability, with mean scores of 21.11, 18.16, and 17.65, respectively [52]. A 2021 qualitative study in Norway of 23 elderly breast cancer survivors 7–8 years post-treatment found that they experienced health issues such as sleep disturbances and pain. Variations in meaning-making were concentrated in six themes, and they used multiple coping strategies to adjust global meaning in order to perceive their cancer experience positively [53].

### 4.3. Characteristics of Effect Patterns

#### 4.3.1. Actor Effect: The Influence of Individual Factors on One’s Own Outcomes

The actor effect represents the primary pattern underlying the association between dyadic coping and illness uncertainty, meaning that an individual’s dyadic coping strategies or perceived uncertainty mainly influence their own physical and psychological outcomes. A 2023 APIM analysis of 484 advanced cancer patient–family caregiver dyads in the United States demonstrated that the negative impact of patients’ illness uncertainty on their own quality of life was significantly stronger than that on caregivers’ quality of life [23].

Two Chinese studies conducted in 2023 supported the actor effect. An APIMeM analysis of 277 pairs of patients with pancreatic cancer and their spouses showed that dyadic intimacy positively predicted individuals’ own dyadic coping, and dyadic coping negatively predicted their own anxiety and depression. A study of 200 pairs of patients with colorectal cancer and their spouses revealed that patients’ self-perceived burden positively predicted both their own and their spouses’ anxiety [25,54].

A 2025 study in South Korea indicated that the psychological status of caregivers of colorectal cancer patients exerted a direct negative effect on patients’ self-care ability. In addition, both partners’ mental health exerted a significant negative effect on their own quality of life (actor effect) [12].

#### 4.3.2. Partner Effect: The Cross-Individual Influence of One Person’s Factors on the Other’s Outcomes

Although the partner effect is weaker than the actor effect, it remains meaningful in specific contexts (e.g., advanced cancer, high caregiving burden), meaning that patients’ or caregivers’ dyadic coping or uncertainty exerts cross-individual influences on the other’s physical and psychological outcomes. A 2025 study from South Korea reported significant mutual influence (partner effect) between the mental health of colorectal cancer patients and their family caregivers [12]. A 2023 APIM analysis in the United States revealed that illness uncertainty among patients with advanced cancer (stage III or IV) had a significant negative partner effect on caregivers’ quality of life [23]. A 2023 Chinese study of 443 lung cancer survivor–spouse dyads found that unmet supportive care needs among lung cancer survivors exerted a significant partner effect on spouses’ quality of life [16].

The direction and strength of the partner effect are also influenced by coping styles. A 2022 study of 566 advanced cancer patient-partner dyads in the Netherlands showed that partners’ ‘supportive dyadic coping’ exerted a negative partner effect on patients’ emotional functioning, whereas ‘negative dyadic coping’ had a positive partner effect on patients’ emotional functioning [28]. A 2024 APIM analysis of 177 cervical cancer patient-spouse dyads in China found that spouses’ ‘positive dyadic coping’ had a significant positive partner effect on patients’ psychological resilience, while negative dyadic coping showed no significant partner effect on patients’ psychological resilience [55].

The magnitude of actor and partner effects varies substantially by cancer type, disease stage, and cultural context, as further analyzed in the heterogeneity section.

Notably, findings regarding the significance of partner effects show inconsistencies across studies. For example, a South Korean study of mixed-cancer patient-family dyads did not detect significant partner effects of unmet supportive care needs on quality of life for either member of the dyad [36], whereas a Chinese study of lung cancer survivor-spouse dyads identified significant negative partner effects of survivors’ unmet needs on spouses’ quality of life [16]. These discrepancies are likely attributable to differences in cancer type, disease stage, and caregiver relationship type across samples, indicating that cross-partner effects are context-dependent rather than universal.

Notably, most evidence supporting these pathways comes from cross-sectional designs, which cannot establish causal temporality or verify the proposed vicious cycle. Future longitudinal studies using cross-lagged panel models, daily diary designs, or chained mediation APIM analyses are needed to empirically test the bidirectional cyclic relationship between the two constructs. Most studies also rely on self-report scales, which may introduce recall bias and social desirability bias.

## 5. Analysis of Heterogeneous Characteristics Across Different Contexts

### 5.1. Heterogeneity by Cancer Type

#### 5.1.1. High-Incidence Cancers: Distinctive Characteristics of Breast, Prostate, and Lung Cancer

**Breast cancer:** Dyadic coping focuses on body image distress and sexual health, while uncertainty mainly stems from ‘risk of recurrence’ and ‘treatment side effects’. For sexual adaptation and intimate relationship outcomes, affectionate behaviors such as emotional intimacy and cuddling act as protective factors for maintaining sexual activity during chemotherapy, as demonstrated by a large Danish longitudinal cohort of 722 patient-partner dyads [56]. Regarding uncertainty-related psychological outcomes, intrusive rumination has been identified as a partial mediator between illness uncertainty and fear of cancer recurrence among Turkish breast cancer survivors [57]. Results from latent profile analysis also indicate that family adaptation among breast cancer patients can be categorized into three latent classes: low, moderate, and high. A positive association exists between the level of dyadic coping and family adaptation, while perceived stress, benefit finding, monthly personal income, cancer recurrence status, and other factors jointly determine the classification of adaptation subgroups [58]. This suggests that interventions for breast cancer patients and their partners should emphasize enhancing dyadic coping skills, stress regulation, and cognitive restructuring, while also accounting for individual differences such as economic conditions and history of cancer recurrence.

**Prostate cancer:** Dyadic coping focuses on the management of treatment side effects (e.g., urinary incontinence, erectile dysfunction), while uncertainty mainly stems from treatment decision-making and sexual function recovery. A 2024 secondary analysis of a randomized clinical trial of 119 prostate cancer patients in Canada showed that a 6-month home-based PC-PEP video intervention significantly improved patients’ emotional well-being at 12 months (FACT-P scale) and significantly increased their support group participation [59]. An e-health intervention for prostate cancer patient–partner dyads, implemented during the COVID-19 pandemic, achieved an enrollment rate of 85.11% and a 12-month retention rate of 78.93% through rapid case identification via cancer registries, a mixed online and telephone study design, and multiple trust-building communication strategies. Further, differential characteristics—including higher dropout risk among non-White participants and greater preference for telephone surveys among older participants—confirmed the association between population characteristics and intervention engagement outcomes [60].

**Lung cancer:** Dyadic coping focuses on symptom management (e.g., dyspnea, pain) and prognostic adaptation, while uncertainty mainly arises from the rate of disease progression and fluctuations in treatment response. A 2022 APIM analysis of 182 postoperative lung cancer patient-caregiver dyads in China showed that patients had a higher risk of depression (34%) than caregivers (19.2%). Caregiver depression not only negatively affected their own intimate relationship quality but also exerted a negative influence on patients’ intimate relationships [38]. A 2021 U.S. study of 522 older advanced cancer patients aged 70 years and older revealed that, among dimensions of illness uncertainty, items related to treatment and physician-patient communication had a stronger negative impact on patients’ psychological outcomes (anxiety, depression, etc.) than uncertainty concerning prognosis [9].

#### 5.1.2. Rare Cancers: Unique Challenges Under Special Disease Trajectories

Uveal melanoma: Uncertainty stems from ‘limited clinical experience due to rarity’ and ‘fluctuating responses to immunotherapy.’ An international qualitative study published in 2025 showed that among patients with metastatic uveal melanoma receiving novel immunotherapy or targeted therapy, illness uncertainty mainly arose from the rarity of the disease, the novelty and complexity of treatment regimens, and “scanxiety” associated with regular scanning to evaluate treatment response. The study also found that patients used metacognitive strategies and strived to maintain daily routines to cope with uncertainty, yet these experiences still triggered significant anxiety [21].

**Cutaneous T-cell lymphoma (CTCL):** Uncertainty centers on the reciprocal effects between pregnancy and disease progression. A 2022 retrospective study in the United States of 22 pregnant patients with early-stage cutaneous T-cell lymphoma showed that 56.8% experienced disease progression during pregnancy, 32.4% remained stable, and 10.8% improved. Pregnancy-related immunosuppression and treatment discontinuation may exacerbate the disease. In addition, limited safe therapeutic options during pregnancy have led some patients to forgo pregnancy in order to maintain treatment [61].

**Adenomyoepithelioma of the breast (AME):** Uncertainty arises from ‘diagnostic difficulty’ and ‘lack of consensus on treatment protocols’. A 2022 UK case report of AME described an extremely rare presentation in an elderly woman in her 80s, whose care was delayed due to COVID-19 lockdown restrictions, resulting in a large edematous and ulcerative lesion in the right breast. Eventually, due to clinical deterioration, an emergency procedure was performed following multidisciplinary team (MDT) evaluation and decision-making. The patient underwent emergency mastectomy with axillary sampling and recovered well postoperatively. The substantial uncertainty encountered during the diagnostic process underscores the necessity of multidisciplinary collaboration and flexible management pathways [62], but cannot be generalized to broader populations due to the case report design. For rare cancer populations with limited sample sizes, individualized precision interventions, technology-assisted asynchronous interventions, and multi-center collaborative study designs represent feasible directions for future intervention research.

### 5.2. Heterogeneity in Population Characteristics

#### 5.2.1. Age Dimension: Differences Among Young, Middle-Aged, and Older Dyads

Young dyads (aged 18–39): Core challenges include ‘childcare responsibilities’, ‘career development’, and ‘concerns about reproductive health’. Dyadic coping coordination is relatively low, and uncertainty has a stronger impact on psychological distress. A 2025 qualitative study of 15 young adult cancer couples in the United States found that they faced challenges in dyadic communication, relationship changes, social support networks, and resource needs, and required age-appropriate interventions and support [51]. A 2022 U.S. study adapting interventions for partners of young adult cancer patients aged 18–39 identified prominent reproductive and sexual health concerns in this group: female patients commonly feared infertility caused by cancer treatment, and couples frequently reported sexual health problems [63]. A 2021 grounded theory study of 13 young adult U.S. patients with advanced cancer aged 23–38 revealed that patients experienced unique uncertainty specific to their life stage, which exacerbated feelings of lost self-identity and triggered profound psychological distress [64].

Middle-aged dyads (aged 40–64): Core challenges include ‘multiple role stressors’ (e.g., caring for both parents and children) and adaptation to disease prognosis. Dyadic coping is mainly ‘problem-solving oriented’, with relatively stronger capacity to regulate illness uncertainty. A 2022 exploratory study of 49 cancer survivor couples aged 27–58 in the United States showed that 37% of survivors and 27% of partners met clinical criteria for depression. Age and sex of survivors moderated the association between dyadic coping behaviors (such as protective buffering) and depressive symptoms [65]. A 2023 study conducted in Jinzhou, China, using latent profile analysis among 254 breast cancer patient–spouse dyads identified four latent classes of dyadic coping patterns, among which the ‘high-level coping group’ accounted for the largest proportion (51.2%). The study further found that couples in the high-level coping group had significantly higher post-traumatic growth scores (PTGI scale) than those in other coping groups [50].

Older dyads (aged ≥65 years): Core challenges include ‘comorbidity burden’ and declining caregiving capacity. Dyadic coping is primarily ‘emotional support-oriented’, and perceived uncertainty declines gradually over time. A 2022 longitudinal study of 397 advanced cancer patient-caregiver dyads aged ≥70 years in the United States showed that uncertainty among both patients and caregivers decreased over time. Moreover, caregivers receiving geriatric assessment interventions experienced a greater reduction in uncertainty than those in the usual care group [18]. A 2023 survey of 112 lung cancer patients aged ≥65 years in South Korea found that the mean self-efficacy score among older patients was 6.04 out of 10. Self-efficacy and appraisal of uncertainty (threat/opportunity) were major predictors of quality of life, explaining 74.2% of its variance [46].

#### 5.2.2. Relationship Type: Differences Among Spousal, Parent–Child, and Other Relative Caregivers

**Spousal caregivers:** The core of dyadic coping lies in ‘intimate relationship maintenance’ and ‘shared decision-making’, with a more pronounced partner effect. A 2023 APIMeM analysis of 277 pancreatic cancer patient–spouse dyads in China showed that patients’ intimate relationship quality (QRI scale) exerted a significant total negative effect on spouses’ anxiety and depression [25]. In a 2022 Chinese study of 254 family caregivers of patients with advanced lung cancer, illness uncertainty was positively correlated with anticipatory grief, and caregiving burden was identified as a significant predictor of anticipatory grief [10].

Notably, a 2024 study conducted in the United States indicated that ‘dyads of advanced cancer patients with minor children and their spouses’ exhibited even more pronounced intragroup differences. Spouses reported significantly higher levels of parenting concern (measured by the PCQ) and anxiety than patients themselves. Parenting concern was significantly negatively associated with quality of illness communication (assessed via the CICS) and family functioning (measured by the FRI). Furthermore, relationship functioning only alleviated depressive symptoms among individuals with low parenting concern, whereas those with high parenting concern maintained elevated depressive levels regardless of relationship functioning [66]. This finding further elucidates the unique stress process experienced by spouses in the dual role of ‘patient caregiver’ and ‘minor child caregiver’, while also expanding the understanding of specific dimensions of intragroup heterogeneity among spousal caregivers.

A 2025 study conducted in China indicated that breast cancer patients aged 50 years or younger tended to evaluate spousal care from a ‘person-centered perspective’, whereas their male spouses focused more on a ‘task-oriented framework’ (emphasizing a sense of control during supportive care). This divergence in evaluative frameworks may result in patients perceiving that their needs are not being fully met [67].

**Parent–child caregivers:** The core of dyadic coping centers on ‘intergenerational role reversal’ (e.g., adult children caring for parents) and ‘surrogate medical decision-making’. Uncertainty mainly stems from difficulties in interpreting parents’ preferences. A 2021 qualitative study in Germany involving 37 older cancer patients and 34 caregivers found that 86% of patients had caregiver support, yet only 38% of caregivers participated in physician-patient consultations. Participation was higher among ‘active caregivers’ (45%) than ‘passive caregivers’ (31%) [68]. Interview data revealed that in some parent–child care relationships, caregivers (e.g., children) actively intervened in treatment decisions when they believed the parents’ choices were inconsistent with their longstanding values.

**Other relative caregivers:** The core of dyadic coping involves ‘legitimacy of the caregiving role’ and ‘coordination of the family support network,’ while uncertainty primarily arises from ‘ambiguous boundaries of caregiving responsibility.’ A 2021 qualitative study of family caregivers of older cancer patients in Germany showed that caregiver participation varied by relationship type: spousal caregivers were more likely to form a joint decision-making unit, whereas other relative caregivers (e.g., adult children) often assumed a more active role amid reversed caregiving relationships, yet their participation in physician-patient consultations remained low (only 38%). In contexts of ‘reversed caregiving relationships’, non-spousal caregivers (e.g., adult children) engaged proactively and intensively in critical physician-patient communication to ensure that older patients comprehended information and could participate in treatment decisions [68].

#### 5.2.3. Cultural and Social Contexts: Differences Between Chinese and Western Societies

Cultural differences between China and the West: Chinese pairs of patient and caregiver usually focus on “family-centered coping together,” while pairs from Western cultures pay more attention to “keeping a good balance between personal freedom and the support that a partner gives.” A 2023 study done in China worked with 443 lung cancer survivor and spousal caregiver pairs, and used the APIM model to look at their data. It found that the number of unmet supportive care needs both people had had a strong noticeable effect on their own quality of life. Patients’ unmet needs also exerted negative partner effects on spouses’ quality of life. This pattern of stronger cross-partner influence aligns with the collectivist cultural context of shared family responsibility, differing from Western samples where actor effects tend to dominate. This shows that the two people in the pair depend on each other when they manage the disease. The study recommended implementing ‘needs-driven family collaborative care interventions’ [16]. A 2021 intervention study from the US called “Me in We” worked with 13 pairs of advanced cancer patients and their caregivers. The study found that the project pushed participants to talk about both individual goals and goals they shared, and this helped improve communication between the two people in each pair [42]. A 2022 study done in China looked at 128 patients who just got diagnosed with breast cancer, and it talked about illness uncertainty. The study found that younger patients said they felt more uncertainty than older ones, and people who had a religious connection were linked to having much lower levels of this uncertainty [69].

In 2021, researchers ran a randomized controlled trial with 224 former cancer genetic counselors. They found that how counselees acted as individuals changed how much uncertainty communication strategies affected people’s emotional and thinking-based results [31]. The Bayesian logistic LASSO regression model worked about the same as the traditional LASSO model when we looked at predicting the risk of acute care use within 30 days for people getting chemotherapy. At the same time, it lets us properly measure how uncertain the predictions actually are. This uncertainty changed quite a bit between different patient groups that are split up by race, what type of cancer they have, and how far along their disease is [70].

#### 5.2.4. Differences in Comprehensive Characteristics Related to Personal Attributes, Resource Conditions and Dyadic Interaction

A cross-sectional study of 254 lung cancer patient–family caregiver dyads in China showed that patients’ illness perceptions positively predicted their depression and anxiety scores, whereas mindfulness and self-compassion negatively predicted these scores; in addition, living with adult children was negatively associated with anxiety. For caregivers, illness perceptions and the number of other available caregivers positively predicted depression and anxiety, while dyadic coping and urban residence negatively predicted these outcomes; self-compassion only negatively predicted anxiety [71]. International research has also confirmed heterogeneity in prognostic cognition and needs among dyads: up to 25% of advanced cancer patient–caregiver dyads show discordance in prognostic information preferences or perceptions. This discordance is even more likely when patients have better physical function and when dyads already differ in their preferences for prognostic information [72].

### 5.3. Heterogeneity Across Disease Stages

#### 5.3.1. Newly Diagnosed Phase (0–3 Months After Diagnosis): Peak Uncertainty and Initiation of Coping

The newly diagnosed phase represents the ‘peak of illness uncertainty.’ Dyadic coping is dominated by ‘information seeking’ and ‘emotional comfort,’ with relatively weak coping capacity. A 2021 cross-sectional study of 115 cancer patient–family dyads in South Korea found that both patients and family members reported high levels of unmet needs, especially in the domain of information [36]. A 2021 cross-sectional study of 84 advanced cancer patient–spousal caregiver dyads in the United States revealed that within structured problem-solving discussions, caregivers’ hostile communication was positively associated with their self-reported subjective burden [35]. A 2024 Chinese study of 202 newly diagnosed cancer patients found that the prevalence of fear of cancer progression during this stage reached 87.62% [49].

#### 5.3.2. Treatment Phase (3 Months to 2 Years After Diagnosis): Adjustment of Coping Strategies and Differentiation of Uncertainty

During the treatment phase, dyadic coping adapts to treatment progress, and uncertainty gradually diverges depending on treatment response. In a 2021 US feasibility study of prostate cancer patients undergoing radiotherapy and their spouses, 10 couples were enrolled and 8 completed the intervention. Mean adherence to the ‘Exercising Together^©^’ dyadic resistance training program during radiotherapy was 83%. After the intervention, patients showed significant improvements in gait speed and functional capacity, as well as reduced anxiety, while spouses exhibited significant enhancements in the Short Physical Performance Battery [73]. A 2023 APIMeM analysis of 277 pancreatic cancer patient–spousal caregiver dyads in China found that intimate relationship quality exerted significant positive predictive effects on dyadic coping: patients’ own intimate relationship positively predicted both their own dyadic coping and their spouses’ dyadic coping, and spouses’ own intimate relationship positively predicted their own dyadic coping [25].

A 2022 prospective study in Spain involving 508 patients with advanced cancer showed that 36.4% of patients experienced illness uncertainty, which was significantly higher among those with comorbid chronic or psychiatric conditions and those with poorer ECOG performance status [74]. A secondary analysis of 46 patients with early-stage ER+ breast cancer, published in the US in February 2022 and available on PMC, found that patients with low 21-gene recurrence scores (RS) had significantly lower overall quality of shared decision-making than those with high RS [75].

#### 5.3.3. Rehabilitation/Advanced Stage (After Treatment Completion or Stage IV): Long-Term Coping and Adaptation to Uncertainty

**Rehabilitation Phase (≥6 months after treatment completion):** Dyadic coping focuses on ‘recurrence surveillance’ and ‘lifestyle adjustment,’ with uncertainty mainly stemming from ‘risk of cancer recurrence.’ A 2022 Chinese study of 231 breast cancer survivors found that fear of cancer recurrence was positively correlated with illness uncertainty, and social support indirectly alleviated fear of recurrence by reducing illness uncertainty [76]. A 2021 qualitative study in Norway of 23 older breast cancer survivors aged 60–86 identified six major themes regarding variations in personal meaning. Common coping strategies included social comparison, positive reappraisal, denial, problem-focused coping, and valuing everyday events [53].

**Advanced Stage (Stage IV or incurable):** Dyadic coping focuses on palliative care decision-making and meaning-making, with uncertainty primarily arising from predicting time to death and maintaining quality of life. A 2023 US APIM analysis of 484 advanced cancer patient–caregiver dyads showed that illness uncertainty among advanced patients exerted significant negative effects on both their own quality of life and that of their caregivers [23]. In a UK qualitative study conducted between 2010 and 2012 and published in 2021, 22 patients with inoperable lung cancer received direct, clear prognostic communication with clinicians [77]. This communication enabled patients and informal caregivers to develop a clear understanding of their diagnosis and prognosis.

Existing heterogeneity studies are mostly exploratory, with inconsistent adjustment for confounding variables across studies, which limits the comparability of findings.

## 6. Development Status and Innovative Direction Suggestions of Integrated Dyadic Interventions

### 6.1. Types and Effects of Existing Interventions

#### 6.1.1. Communication-Oriented Interventions: Focusing on Dyadic Information Exchange and Decision Coordination

Communication-oriented interventions reduce informational ambiguity and alleviate illness uncertainty by training dyads in effective communication skills. A 2025 U. S. study ran a four-step program to give parental support to pairs made up of advanced cancer patients who have minor children and their spouses. The program covered four main areas: talking about the illness, managing daily family life, helping caregivers, and getting ready for end-of-life care. The program showed good results for how easy it was to run and how well people accepted it. When researchers checked back in after six weeks, the group that got the intervention had much lower anxiety symptoms, and their confidence in their parenting skills got better too [78].

When it comes to making real progress in communication-focused interventions, we already have clear proof that interventions targeting specific types of cancer do work well. A research study done in China in 2025 looked at pairs of prostate cancer patients and their informal caregivers, and it found that nursing interventions that work with the whole dyad helped patients get better at dyadic coping. At the same time, these interventions cut down on the anxiety that the caregivers feel and improved both the caregivers’ self-efficacy and the patients’ sexual function. Also, interventions that run less than three months and use a mix of delivery formats helped lower caregiver anxiety more. At the same time, when interventions are delivered in person, they show clear improvement in patients’ relationship satisfaction [79]. What these results show is that when we do communication-focused interventions, making one-on-one interaction, working together and personalized delivery methods work better can actually deal with the main problems that come up when patients adjust to their disease. This gives useful ideas for interventions that work with similar pairs of cancer patients and their partners.

#### 6.1.2. Cognitive Behavioral-Oriented Interventions: Integrating Uncertainty Restructuring and Coping Skills Training

Cognitive behavioral-oriented interventions simultaneously improve cognitive and behavioral outcomes in dyads through a dual module of “cognitive restructuring plus behavioral training.” A 2025 quasi-experimental study conducted in China among patients with glioma undergoing chemotherapy indicated that patients receiving cognitive behavioral therapy exhibited significantly lower scores for illness uncertainty, the impact of stressful life events, anxiety, and depression after four cycles of chemotherapy compared with those receiving only routine care, while their quality of life was also significantly higher. This study confirms that cognitive behavioral-oriented interventions are effective in reducing illness uncertainty and psychological distress and improving quality of life in this patient population [80]. Virtual reality-assisted cognitive behavioral therapy extends traditional CBT by enabling immersive, controlled exposure to personalized stress triggers and rehearsal of coping strategies, with transdiagnostic mechanisms relevant to anxiety and depression, although the current evidence base remains preliminary and methodologically heterogeneous [81].

A three-arm randomized controlled trial published in the United States in 2023 enrolled patients with glioma undergoing radiotherapy and their family caregivers, who were assigned to an individual yoga group, a dyadic yoga group, and a usual care group for a 6-week intervention. The results showed that caregivers in the individual yoga group achieved greater improvements in subjective benefits, psychological quality of life, financial burden, caregiver self-esteem, and health decline. The dyadic yoga group showed no significant advantages over the usual care group, and there were no significant between-group differences in depressive symptoms across the three groups [44]. A multicenter observational study conducted in the Netherlands in 2022 involving 566 dyads of advanced cancer patients and their partners demonstrated that negative dyadic coping (measured by the DCI scale) exhibited significant positive actor effects and partner effects between patients and partners. In addition, patients’ illness perceptions were associated with the emotional functioning of both partners [28].

#### 6.1.3. Technology-Assisted Interventions: Expanding Intervention Accessibility Through Digital Tools

Technology-assisted interventions address the “spatiotemporal limitations” of traditional interventions by using digital tools such as mobile applications and remote monitoring, which are especially suitable for dyads with heavy caregiving burdens or poor transportation access. The ‘MGCS mobile health intervention app,’ tested in China between 2021 and 2022, was applied to 168 newly diagnosed gynecologic cancer patients aged 18 years and older undergoing chemotherapy. The app was split into four different modules: “weekly themes,” “emotional support,” “discussion forum,” and “health consultation.” After 24 weeks of the intervention program, the illness uncertainty scores for patients (which we measured with the MUIS-A scale) went down a good amount. On average, people logged in around 2 to 3 times every week, and the total number of logins across all 24 weeks was about 67.9 [19]. A 2025 French study looked at a technology-supported combined method for diagnosing prostate cancer. The study used a dual-mode deep learning model that combined clinical data with biparametric magnetic resonance imaging. When compared to single-mode models, this one got much better diagnostic results. It also lets people tell different levels of prediction confidence apart by using uncertainty measures like predictive entropy, and this helps cut down how much work clinicians have to do [82].

Beyond mobile health apps, virtual reality-assisted cognitive behavioral interventions represent an emerging direction for future dyadic care, as they can deliver immersive, standardized coping skills training while reducing geographic access barriers.

Despite their potential advantages, technology-assisted dyadic interventions still face non-negligible implementation risks. First is the digital divide: elderly dyads and those with low education levels may have difficulty using mobile apps or VR devices independently, which may instead widen gaps in health resource access. Second is the adherence dilemma: existing digital interventions generally show polarized usage, with high engagement in a small number of dyads and continuous dropout in most dyads in the middle and late stages. Third is the risk of information overload: unfiltered health information may further amplify rather than alleviate illness uncertainty, especially for dyads with low health literacy.

Consistent with evidence from cancer dyads, exploratory cross-sectional research in other chronic stress contexts has also confirmed the robust association between behavioral patterns and mental health outcomes, which further validates the theoretical foundation of cognitive-behavioral intervention frameworks across different populations.

### 6.2. Common Limitations of Existing Interventions

Even though combined dyadic interventions have gotten some positive results, studies done between 2021 and 2025 have found important limitations in five different areas.

#### 6.2.1. Not Enough Representative Samples and Weak Generalizability

A number of current intervention studies center on prostate cancer, but work on rarer cancers like uveal melanoma is still pretty limited. Some existing studies on this topic are non-intervention explorations, while other studies are intervention studies conducted in stable or updated treatment contexts [21,73]. Among older adult patients with advanced cancer and their caregiver dyads, 63.98% of caregivers had some college education or higher [18], making it difficult to generalize intervention effects to diverse populations.

#### 6.2.2. Not Enough Intervention Time and No Clear Long-Term Results

Most existing interventions only do follow-up checks for three months or less. Just a few studies keep tracking outcomes that go over six months. As one example, the “Me in We” program from the US has already shown that it works pretty well and is easy to accept for people with late-stage cancer and the family members who care for them. However, this study only collected and reported data from post-intervention evaluations conducted immediately after the two intervention sessions, with no additional follow-up data collected or reported beyond these sessions [42]. The Chinese MGCS mobile app intervention only included a 24-week follow-up, which cannot verify the sustained effects of long-term use [19]. For cognitive-behavioral dyadic interventions, a minimum of 3 months of follow-up is recommended to confirm short-term maintenance of coping skills, and 6 months or longer is needed to verify sustained long-term effects on illness uncertainty and dyadic outcomes.

#### 6.2.3. Insufficient Integration of Comprehensive Factors

Not enough different kinds of factors are put together in the research right now. Just a small number of interventions already think about different traits like what type of cancer it is and what cultural background people come from; most of the existing work still uses a “one-size-fits-all” design that does not adapt to differences. For example, the Western-developed CoupleLinks online intervention targets young couples affected by breast cancer, with core features of convenience, mutual participation, and professional facilitator guidance, yielding high overall satisfaction [43]. However, it was not adapted to the cultural characteristic of “family involvement in medical decision-making” in China, resulting in significantly lower participation rates among young Chinese breast cancer couples compared with Western counterparts. Interventions based on geriatric assessment include comorbidity screening but do not explicitly mention enhanced management modules for comorbidities. In addition, the main reason for dropout among dyads was associated with patients entering hospice care or death [18].

#### 6.2.4. Imbalance Between Practicality and Accessibility in Technological Interventions

Although technology-assisted interventions expand accessibility, they suffer from problems of “functional redundancy” and the “digital divide.” For example, the MGCS mobile application comprises four modules (weekly topics, emotional support, discussion forum, and health consultation) for Chinese gynecologic cancer patients undergoing chemotherapy. It has been verified in a 24-week multicenter randomized controlled trial that the app can significantly reduce patients’ illness uncertainty and improve quality of life, while its usage shows polarization in login frequency and duration [19]. To mitigate the digital divide for elderly or low-literacy dyads, future technology-assisted interventions should incorporate simplified interfaces, voice interaction, multimodal content presentation, and paired offline guidance as core design features.

#### 6.2.5. Deficiencies in the Evaluation System

The design of intervention protocols and assessment procedures has not fully accounted for the impacts of intervention-related adjunctive measures on the natural course of disease and outcome determination, reflecting an inadequate intervention evaluation system. In a 2024 Canadian study on immediate lymphatic reconstruction for breast cancer-related lymphedema, supportive care measures such as early compression therapy altered the natural course of lymphedema and affected the valid assessment of the effects of immediate lymphatic reconstruction. This finding illustrates the unique disturbance to the natural history of disease caused by measurement and intervention in clinical research, analogous to the uncertainty principle [83].

#### 6.2.6. Under-Addressed Practical Risks and Ethical Considerations

From a practical application perspective, existing dyadic interventions also have under-addressed potential risks. Most intervention protocols do not set exclusion criteria for dyads with poor baseline relationship quality, and forced joint coping training may exacerbate relationship conflict and psychological distress. In addition, interventions requiring joint participation of both parties may increase the time burden of overloaded caregivers, and the existing literature lacks research on adverse events and termination mechanisms of dyadic interventions.

Furthermore, most existing interventions are designed for feasibility testing rather than efficacy confirmation, with small sample sizes and short follow-up periods; few studies explicitly map intervention components to changes in both dyadic coping and illness uncertainty through theory-driven evaluation.

### 6.3. Directions for Intervention Innovation

Based on preliminary empirical evidence from existing small-sample intervention studies, the following conceptual directions are proposed for future integrated dyadic interventions, which require validation in large-scale high-quality randomized controlled trials.

#### 6.3.1. Personalized Precision Intervention Design

Step-by-step guidelines can be developed based on cancer type, population characteristics and disease stage, supported by preliminary evidence from targeted interventions in pancreatic and breast cancer populations. For instance, when looking at pairs of people with pancreatic cancer (which has a bad outlook and lots of unknowns) and their spouses, we can add special training and mental support sections. These suggestions are intended to help patient–spouse pairs improve their joint coping with the disease [20]. For Chinese breast cancer survivors, effective interventions targeting social support and illness uncertainty may be explored to alleviate fear of cancer recurrence [76]. When patients are in the hospital for advanced lung cancer, their nurses can give useful help both to the patients and to their spouses. This help usually comes through in-person talks, health lessons, and even mobile digital tools, and it covers basic info about caring for the disease as well as how to handle tough mental situations. This can enhance the couple’s ability to cope with advanced lung cancer, reduce spousal caregiving burden and psychological distress, further maintain healthy family functioning, and strengthen patients’ sense of meaning in life [84].

#### 6.3.2. Cross-Cultural and Family System Adaptation

We need to improve intervention frameworks by adding details about family structure features that differ from culture to culture. To give an example, when working with pairs of Chinese lung cancer survivors and their spouse caregivers, healthcare workers should use customized dyadic family management interventions. This way, they can do a better job of matching the care support needs of both people in the pair [16]. For multiethnic cervical cancer patients and their spouses in Xinjiang, targeted interventions related to dyadic coping and psychological resilience are needed [55]. For dyads of Western advanced cancer patients and family caregivers, balancing individual needs and spousal support can be achieved by guiding the identification and discussion of personal and shared goals [42].

#### 6.3.3. Technology-Humanistic Integrated Interventions

First, the MGCS app should keep its main supportive features and make its functions easier to use and its content clearer, so that it can fit what elderly patients and those with less education actually need. Second, it also needs to offer face-to-face care and help for patients who are getting prostate cancer radiotherapy, as well as their spouses. This help includes group exercise classes led by certified cancer exercise trainers, as well as structured dyadic resistance training that couples can perform together as a team [73]. Meanwhile, clinicians can use validated scales to assess the coping abilities of breast cancer patients and their spouses and deliver positive psychological interventions to strengthen dyadic coping capacity in managing family crises [26].

#### 6.3.4. Long-Term Follow-Up and Effect Translation Mechanisms

Implement dyad-oriented interventions for patients with advanced cancer and their family caregivers to reduce illness uncertainty and improve quality of life for both members [23]. Subsequently, the applicability, feasibility, acceptability, satisfaction, and actual effects of dyadic mindfulness-self-compassion interventions will be examined to explore their potential for widespread use in cancer-related healthcare settings. Intervention effects will focus on key indicators, including anxiety and depressive symptoms, illness perception, and mindfulness status among lung cancer patients and their families [85].

The dual-target intervention framework integrating uncertainty management and dyadic coping skills training is potentially transferable to other chronic diseases (e.g., cardiovascular disease, chronic respiratory disease), where patient-caregiver dyads similarly face long-term uncertainty and collaborative coping needs.

Nevertheless, the transferability of this framework has clear boundary conditions. For chronic diseases with relatively definite prognosis and standardized treatment pathways (such as stable primary hypertension), the core module of uncertainty cognitive restructuring may have limited marginal benefit. For patient groups mainly cared for by professional institutions rather than family caregivers, the dyadic coping skills training component needs to be reconstructed according to the care scenario. Cross-disease migration also requires verification of the adaptability of measurement tools and intervention intensity and cannot be directly replicated from the oncology setting.

For resource-constrained settings without dedicated psycho-oncology specialists, manualized interventions delivered by trained frontline nurses, digital self-help tools, and stepped-care referral models represent feasible implementation pathways.

## 7. Conclusions

### 7.1. Key Findings

This narrative review addresses the fragmentation of existing research by systematically integrating the bidirectional interactive pathways between dyadic coping and illness uncertainty, mapping heterogeneous characteristics across multiple contexts, and proposing a conceptual framework for integrated dual-target interventions. It synthesizes recent evidence on the mechanism underlying the association between illness uncertainty and dyadic coping among patient–caregiver dyads in cancer care, as well as pathways for integrated interventions. For clinical and research practice, three core take-home messages can be highlighted:

1. Accumulating evidence supports a likely bidirectional association between illness uncertainty and dyadic coping in cancer dyads, mediated by self-efficacy and relationship intimacy, and moderated by cancer type, age, and cultural context.
2. Evidence shows that single-target interventions addressing only uncertainty or dyadic coping yield limited holistic benefits; based on these findings, the authors propose that integrated dual-target designs represent a key direction for future clinical optimization.
3. Future interventions should advance toward personalized precision, cross-cultural adaptation, and technology-humanistic integration to match heterogeneous needs across disease trajectories.

Current evidence suggests a likely bidirectional association between illness uncertainty and dyadic coping: illness uncertainty may impair positive dyadic coping through pathways such as cognitive confusion, while dyadic coping may regulate perceived uncertainty via information integration and emotional support. This conclusion is limited by predominantly observational study designs, high methodological heterogeneity, and the absence of formal quality appraisal across all included studies. This interactive process is moderated by heterogeneous factors including cancer type, age, and cultural background, and demonstrates distinct patterns of actor and partner effects. Regarding heterogeneity, dyads differ significantly in their primary coping focus and sources of uncertainty across ‘cancer types’ (e.g., common cancers vs. rare cancers), ‘population characteristics’ (e.g., age, relationship type, cultural background), and disease stages (e.g., newly diagnosed, treatment, survivorship, or advanced disease). Existing integrated interventions include communication-oriented, cognitive behavioral–oriented, technology-assisted, and multidisciplinary exercise-based approaches. Although these interventions have achieved certain effects, they remain limited in several respects. Future interventions should advance toward ‘personalized precision design, cross-cultural family system adaptation, integration of technology and humanistic care, and long-term follow-up with effect translation’, so as to provide theoretical and practical support for improving the quality of care for cancer dyads.

### 7.2. Limitations and Future Research Directions

Although this review summarizes and integrates research on dyadic coping and illness uncertainty in cancer, several limitations remain. Several methodological limitations of this review should be acknowledged first. (1) Only the PubMed database was searched, which may introduce database selection bias. Specialized databases including PsycINFO, CINAHL and Scopus were not included, which may lead to omission of some psychosocial and nursing-specific studies, particularly qualitative research published in niche journals. The decision to use PubMed alone was based on its comprehensive coverage of core oncology and nursing literature and the need to maintain search consistency; however, the potential for missing relevant evidence from other databases is acknowledged. Future systematic reviews on this topic should expand the search to multiple databases to improve comprehensiveness. (2) Only published English full-text articles were included, which may lead to publication bias as studies with negative findings are less likely to be published. To partially mitigate this bias, we included multiple study designs (cross-sectional, qualitative, cohort) beyond intervention trials and explicitly presented inconsistent findings across studies rather than selecting only positive results. Nevertheless, the potential impact of unpublished negative studies cannot be fully excluded. (3) As a narrative review, subjective interpretation may exist in evidence synthesis, and no uniform quantitative quality assessment was performed across all studies. At the foundational research level, existing studies suffer from inadequate sample representativeness: most focus on common cancers such as breast and prostate cancer and young, highly educated populations, with insufficient coverage of rare cancers (e.g., uveal melanoma), elderly and low-education groups, and special dyads in multicultural contexts (e.g., cross-cultural families), restricting the generalizability of findings. At the intervention research level, most interventions have short follow-up periods (≤3 months) and lack long-term tracking to verify sustained and stable effects. Meanwhile, only a minority of interventions adequately account for heterogeneity; one-size-fits-all designs poorly match the personalized needs of diverse dyads. In technology-assisted interventions, functional redundancy and the digital divide coexist, compromising practicality and limiting accessibility for elderly and low-literacy populations. At the evaluation level, current interventions do not sufficiently control for the confounding effects of adjunctive supportive care on the natural disease course, leading to imprecise estimates of intervention efficacy. Furthermore, few comprehensive measurement tools capture both dyadic interaction and uncertainty, making it difficult to fully assess their interactive effects. These issues warrant further refinement in future research.

## Data Availability

No new data were created or analyzed in this study. Data sharing is not applicable to this article.

## Supplementary Materials

The following supporting information can be downloaded at: https://www.mdpi.com/article/10.3390/healthcare14142098/s1.
