# Supplementary figures and images for: Dyadic Coping and Illness Uncertainty in Cancer Patient–Caregiver Dyads: Interactive Mechanisms, Heterogeneity, and Integrated Intervention Directions—A Narrative Review (2021–2025)
Source: Healthcare (Basel). 2026 Jul 14;14(14):2098. doi: 10.3390/healthcare14142098 (PMC13409783; doi:10.3390/healthcare14142098)

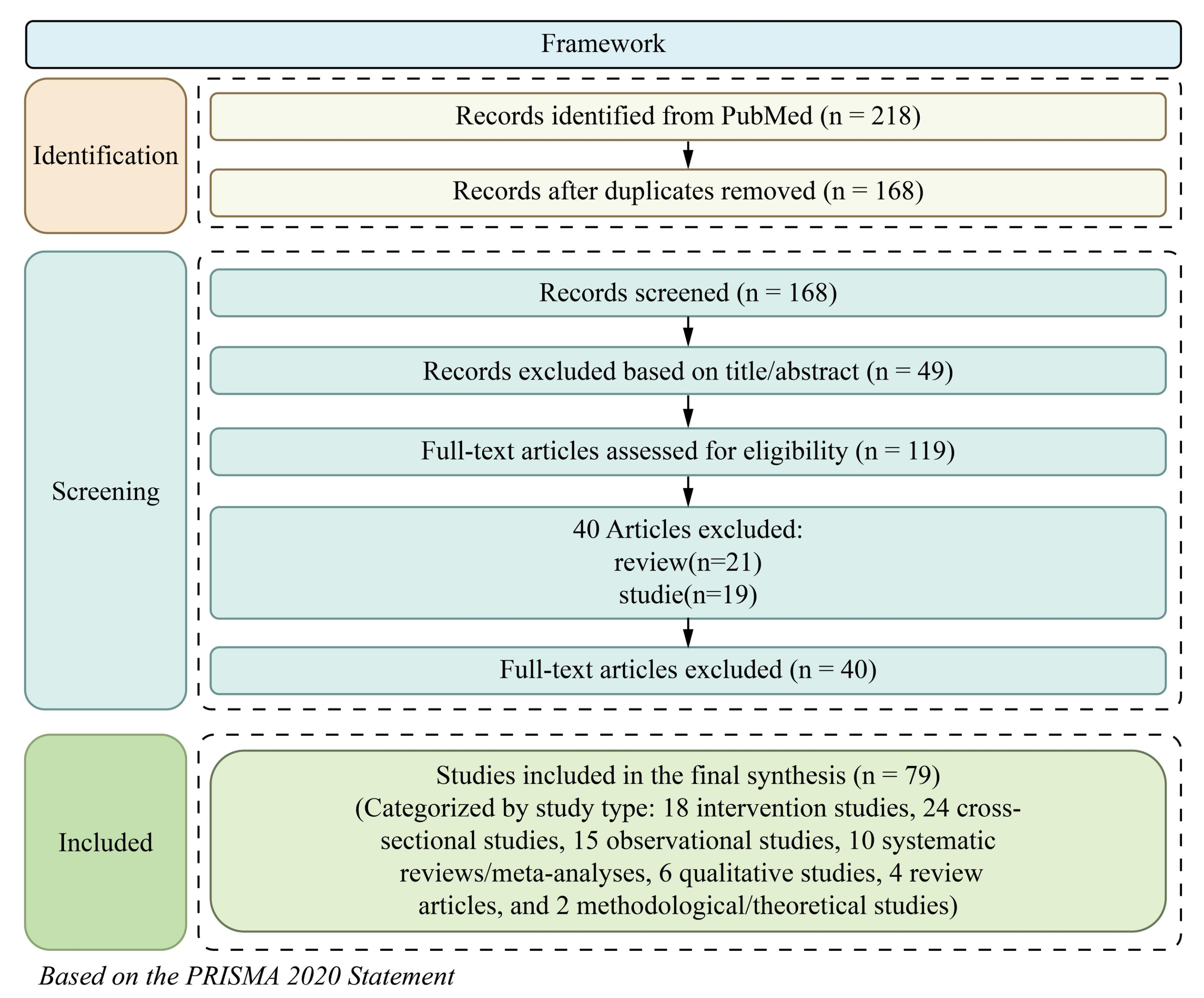

Supplement: Supplementary file 1 [file healthcare-14-02098-s001.zip › healthcare-4377096-supplementary.png]
